# Meaningful work and resilience among teachers: The mediating role of work engagement and job crafting

**DOI:** 10.1371/journal.pone.0222518

**Published:** 2019-09-19

**Authors:** Jessica Van Wingerden, Rob F. Poell

**Affiliations:** 1 Institute of Psychology, Faculty of Social Sciences, Erasmus University Rotterdam, Rotterdam, The Netherlands; 2 Department of Human Resource Studies, Tilburg University, Tilburg, The Netherlands; Universitat de Valencia, SPAIN

## Abstract

Research in the field of work and organizational psychology more and more highlights the importance of employees’ experience of meaningful work. Adding to this area of research, the present study among teachers examined the relationship between meaningful work and resilience and tested whether this proposed relationship is mediated by teachers’ work engagement and job crafting behaviour. Data for this study was collected among a group of Dutch teachers working in a school for primary education (*N* = 174). To test the hypothesized relationships, we conducted a bootstrapping analysis. The outcomes revealed that work engagement and job crafting fully mediated the relationship between meaningful work and teacher’s resilience. The insights provided in this study may be useful for the deliberate cultivation of teachers’ resilience and may help them to stay enthusiastic in their meaningful but demanding profession. Theoretical contributions, limitations, suggestions for future research and practical implications are discussed.

## Introduction

Western society calls for a new generation of teachers to educate children in a century that is characterized by developments such as digitalization and globalization. Teaching is a demanding profession in this era of ‘change’ since teachers are expected to exhibit new skills and dispositions that fit with the developments, which include; problem-solving abilities, technology, collaboration and communication skills [1,2,3]. Teachers working conditions may change in unpredictable ways, and depending on how they experience these changes, they will display resilience or be unable to cope with these changes [4]. Resilience, which can be defined as the process of effectively negotiating, adapting to, or managing substantial sources of stress, [5] may enable teachers to control and impact their changing work environment successfully [6]. One of the factors that can positively influence employees’ resilience is experiencing that one’s work is meaningful [7].

Meaningful work refers to work that is perceived as significant and valuable to an individual [8,9]. Earlier research by Hansen [10] indicated that viewing ones job as meaningful can spark teacher’s resilience with determination and flexibility. Further, research by several scholars revealed that meaningful work is positive related to work outcomes such as work engagement [11–14]. When one feels engaged at work, he or she will be more inclined to increase their job resources and job demands, to create a better suiting and more challenging work environment [15–17]. This proactive behavior by which employees create changes in their work environment and the way they work, is also known as job crafting [18,19]. The outcomes of a study by Lu, Wang, Lu, Du, & Bakker [20], indicated that engaged employees craft their work in physical and relational ways, which helps them to create and/or maintain a good fit between their job and their own talents, passions and preferences at work. Consequently, job crafting can be seen as a strategic advantage one may apply during times of change [21]. Research has revealed that job crafting behaviors are positively associated with several outcomes such as resilience [22,23,15]. All in all, the aim of this study is to examine whether teachers who experience their work to be meaningful are likely to be more resilient, first because they feel engaged at work, and second via their job crafting behavior. As far as we know these proposed relationships have not yet been investigated, even though they could shed a light on how teachers’ resilience may be enhanced/positively influenced.

## Theory and hypotheses development

### Meaningful work and work engagement

Because individuals spend more than a third of their lives at work [24], their identities are often formed in terms of work [25]. Frankl [26] acknowledged that every individual attempts to find meaning in one’s existence, including the experience of meaning in the workplace [27]. Most people want to find a job and/or career that fulfills them in more ways than just making money; they want their job and career to be meaningful [28]. Meaningful work is often valued above work characteristics like job security, promotions, income, or working hours [29]. Meaningful work can be defined as ‘work that is experienced as particularly significant and holding positive meaning for an individual’ [9]. More precise Martela and Pessi [30] argue that meaningful work consists of three components: ‘The subjective experience of work as intrinsically significant and worth doing, the experience that one is able to realize oneself through work, and the work serving a broader purpose’. Meaningful work can be related to fulfilling needs of the self (personal) and/or fulfilling the needs of others (social) [31]. Further, work is experienced as meaningful when the purpose to work exceeds extrinsic outcomes alone (e.g., [32]). The experience of meaningful work, which reflects a deep personal linkage between an employee and his or her work, motivates employees to go above and beyond the normal requirements of their work [33]. The importance of meaningful work for both employees and organizations is underlined by several studies who revealed that meaningful work is positive related to positive personal and work-related outcomes such as effective management of change, retention of key employees, organizational performance, greater organizational commitment, and employee engagement (see [34–40]). Experiencing work to be meaningful may be an important resource for employees to either become or stay engaged at work. Work engagement has been defined as “a positive, fulfilling, work-related state of mind that is characterized by vigor, dedication, and absorption” [41]. Vigor is characterized by having high levels of energy, mental resilience and having the willingness to invest effort in one’s work. Dedication refers to being strongly involved in one’s work, and to feeling a sense of meaning, to be enthusiastic, inspired, feeling pride and challenged by the work. Lastly, absorption is characterized by being concentrated to the fullest, that the time passes by rapidly and one has difficulties with separating oneself from work activities [42].

Experiencing ones work as meaningful is regarded as essential for employees’ work engagement [43,44]. The studies by van Wingerden & van der Stoep [45,46] showed that employees’ experience of meaningful work is positive related to their work engagement. Fouché et al., [7] examined the antecedent and outcomes of meaningful work among school teachers. The outcomes of their study showed that teachers’ experience of meaningful work was positively associated with work engagement. In line with these findings we hypothesize:

*Hypothesis 1*: *Meaningful work is positively related to teachers’ level of work engagement*

### Work engagement and job crafting

Research revealed that employees who are engaged at work, are highly energetic, self-efficacious individuals who exercise control over events that influence their lives [47]. Employees who are engaged at work, will actively change their work environment to maintain their work engagement, if needed [48]. This proactive change behaviour initiated by employees is also known as “job crafting” [49]. Wrzesniewski and Dutton, defined job crafting behavior as the process of employees redefining and reimagining their job designs in personally meaningful ways [49]. Via job crafting employees independently try to alter aspects of their job, to make a more suitable connection between the characteristics of the job and their personal needs, preferences and abilities [22]. Furthermore, job crafting is also recognized as a core element of the JD-R theory [50]. According to the JD-R approach to job crafting, employees could craft their job with four strategies to optimize job demands and job resources [50,15]. First, employees may increase their structural resources at work. An example of this would be that one proactively seeks different tasks which may require innovative skills. Second, employees may proactively increase their social job resources at work. For example choosing with whom one will interact more frequently. Third, employees may increase their challenging demands at work. For example, employees can apply for new projects within the organization. Fourth, employees may try to decrease their job demands, for example by taking more breaks at work. Earlier studies suggested that decreasing hindering job demands is unrelated [1,51] or negatively related to work engagement [52]. Therefore, we will not include the decreasing hindering job demands dimension in the present study.

Earlier research has shown that when one feels engaged, he or she will be more inclined to increase their job resources and job demands, to create a better suiting and more challenging work environment [15–17]. Consequently, employees, who are engaged, are most likely to employ job crafting as strategy to improve their job [14]. Moreover, a study among nearly 750 Finnish managers showed that engaged managers were the most eager to develop themselves in their job and increase their occupational knowledge [53]. Consistent with this idea, Parker & Collins [54] proposed that activated positive affect (e.g. the energy and enthusiasm characteristic of the vigor and dedication dimensions of engagement) promotes proactive action taking. Therefore, employees who are engaged and experience positive affect are more likely to show proactive behavior because they are better able to see possibilities and think innovatively [55,56]. Thus, engaged employees may conserve their own engagement through a process of job crafting. Therefore, the following hypothesis is conducted:

*Hypothesis 2*: *Work engagement is positively related to teachers’ job crafting behavior*

### Job crafting and resilience

As explained in the introduction, resilience can help teachers to cope with the aforementioned developments and manage their work environment successfully [6]. Resilience can be defined as “the capacity to continue to ‘bounce back’, to recover strengths or spirit quickly and efficiently in face of adversity” [57]. Next, according to Sammons et al. [4], resiliency is “a dynamic construct subject to influence by environmental, work-specific and personal contexts” (p. 694). Sammons et al., [4] established that one’s life and working conditions may change in ways that are unpredictable, and that it will depend on ones experiences, perceived competences and views on meaning of engagement whether they will display resilience or be unable to cope with the changes. Tugade and Fredrickson [58] suggest that the ability to find positive meaning in adverse situations and to regulate negative emotions contributes to personal resilience. Therefore, resilience is characterized by positive coping and adaptation in the face of significant risk or adversity [59]. The study by Vogt et al. [60] showed that when people proactively craft a resourceful and challenging work environment for themselves (e.g. job crafting), it can lead to positive outcomes such as resilience. Furthermore, the study by Van Wingerden et al., [51] examined the impact of job crafting among teachers. The outcome of this study revealed that job crafting was a predictor of teachers’ resilience. Thus, job crafting may be a proactive way to enhance teachers’ resiliency. We therefore hypothesize:

*Hypothesis 3*: *Job crafting is positively related to teachers’ level of resilience*

**Fig 1:**
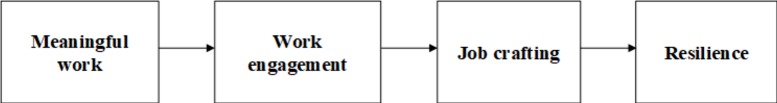
The proposed model.

The theoretical arguments of this study so far suggest that teachers’ experience of their work to be meaningful may be positively related to their work engagement. As suggested, engaged employees may create their own great place to work via job crafting [16]. By doing so, teachers optimize their work environment in a way that it is aligned with their preferences and abilities which subsequently may strengthen teachers’ resilience [61]. Earlier studies revealed evidence for these proposed positive relationships between the variables under study [7,14,42,43,44,59]. However, we not only expect these single relationships between the variables under study as research revealed before. We propose that teachers who experience their work to be meaningful are likely to be more resilient, firstly because they feel engaged to their job, and secondly due to their job crafting behavior (see Fig 1). Thus, we propose that the relationship between meaningful work and resilience is sequentially mediated by work engagement and job crafting. In line with the above, the following is hypothesized:

*Hypothesis 4*: *Meaningful work is positively related to resilience via work engagement and job crafting behavior*

## Method

### Participants and procedure

In this study we collected data among teachers of a Dutch school for primary education using an online survey. The school principle proactively contacted the university to participate in the study on meaningful work among teachers. An e-mail with a link to the questionnaire was sent to all 267 teachers working at the same school. The school principal had sent an informative e-mail to announce the questionnaire and the aim of the study. It emphasized confidentiality and informed the employees that the questionnaire was available for three weeks. A total of 174 employees completed the questionnaire, representing a response rate of 65%. The sample consisted of 151 female (87%) and 22 male (13%) teachers, which are representative percentages for the occupational group [62]. The average age was 45 years (SD 12.01), and 76% of the employees successfully completed a bachelor degree or a master degree. Detailed characteristics of the participants are presented in Table 1.

**Table 1:** Baseline characteristics.

Characteristic	N (%)	Characteristic	N (%)
*Gender*		*Organizational tenure*	
Male	22 (12)	< 2 years	21(12)
female	152 (88)	2–3 years	7 (4)
		4–6 years	9 (5)
*Age*		7–10 years	33 (19)
< 30	17 (10)	11–15 years	44 (26)
30–39	58 (22)	≥16 years	60 (35)
40–49	28 (16)		
≥ 50	74 (42)	*Type of contract*	
		Permanent	153(88)
*Educational level*		Fixed term	15 (9)
Junior secondary education	12 (6)	Other	6 (3)
Senior secondary education	31 (18)		
Bachelor degree	120(70)	*Supervisory position*	
Master degree	11 (6)	Yes	16 (9)
		No	158 (91)

### Measures

*Meaningful work* was measured with the subscale positive meaning of the Work as Meaning Inventory (WAMI) scale [12]. This subscale was chosen as captures the sense that people judge their work to matter and be meaningful. An example item is: I have a good sense of what makes my job meaningful. A Five-point scale was used with answers ranging from 1 (totally disagree) to 5 (totally agree). The internal consistency of the scale was good (α = .81).

*Work engagement* was measured with the nine-item, Dutch version of the Utrecht Work Engagement Scale (UWES; [63]). This version consists of three subscales to assess each engagement dimension: vigor, dedication and absorption. An example of each subscale: “At my job, I feel strong and vigorous” (vigor: α = .78), “My job inspires me” (dedication: α = .76), and “I get carried away when I am working” (absorption: α = .80). Participants could respond to these items using a Seven-point frequency scale ranging from 1 (never) to 7 (always).

*Job crafting* was measured with three subscales of the Job crafting scale [15]. We chose for increasing social job resources, increasing structural job resources and increasing challenging job demands. As mentioned before previous studies suggested that decreasing hindering job demands is unrelated [15,51] or negatively related to work engagement [45]. Thus, the decreasing hindering job demands subscale was not included in the present study. Of each subscale, three items were included and scored on a five-point scale ranging from 1 (never) to 5 (very often). Examples are: “I ask colleagues for advice” (increasing social job resources; α = .72) “I try to develop my capabilities” (increasing structural job resources; α = .74), and “When there is not much to do at work, I see it as a chance to start new projects” (increasing challenging job demands; α = .76). Earlier studies suggested that decreasing hindering job demands is unrelated [15, 51] or negatively related to work engagement [45]. Therefore, we will not include the decreasing hindering job demands dimension in the present study.

*Resilience* was measured using a six item version of the scale developed by Luthans, Avolio, Avey and Norman [64]. An example is “At this moment, I can manage difficulties at work very well”. All of the items were scored ranging from 1 (never) to 5 (always). The internal consistency of the scale was good (α = .82).

### Analysis

First, descriptive analyses were conducted with SPSS [65] (version 23) in which the correlations of all the variables under study were included. A simple regression analysis was performed to test the direct relationship between meaningful work and teachers’ resilience, followed by a serial mediation analysis with PROCESS Macro Hayes model 6 [66]. All indirect effects were subjected to follow-up bootstrap analyses with 5000 bootstrap samples and 95% bias corrected confidence intervals. Serial mediation assumes “a causal chain linking the mediators, with a specified direction of causal flow” [67]. For serial mediation, PROCESS tests all possible variable combinations for a particular variable ordering (specified by the analyst).

## Results

### Descriptive statistics

An overview of the descriptive statistics for all the study variables, including means, standard deviations, correlations, and Cronbach’s alpha can be found in Table 2. Preliminary analyses were carried out in order to test for linearity, homoscedasticity, collinearity and multicollinearity. No violations of assumptions were identified for all the variables under study, before running correlations and model analyses. Pearson’s correlation was used to examine the relationships between the different variables; gender, age, meaningful work, work engagement, job crafting and resilience.

**Table 2:** Summary of the descriptive statistics for all the main variables, including means, standard deviations, correlations, and Cronbach’s alpha.

	M	SD	1	2	3	4	5	6
1. Gender	1.88	.34	-					
2. Age	45.17	12.01	-.08	-				
3. Meaningful work	7.33	1.32	-.02	.03	(0.81)			
4. Work engagement	6.44	1.58	.07	.06	.41**	(0.88)		
5. Job crafting	6.15	1.21	-.04	-.04	.37**	.51**	(0.82)	
6. Resilience	6.56	1.15	-.05	-.06	.23**	.35**	.41**	(0.82)

** p < .01

### Hypothesis testing

**Fig 2:**
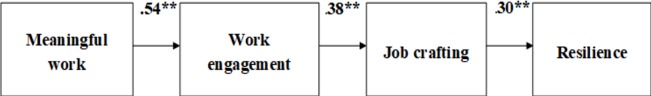
Final results of the proposed model.

A simple regression analysis was used to evaluate the effects of meaningful work on work engagement, the effects of work engagement on job crafting, and lastly the effects of job crafting on resilience. The results from the simple regression analysis showed that there was a significant relationship between meaningful work and work engagement (H1), *b* = .54, SE = .07, *p* < .001. Approximately 27% of the variance in work engagement was accounted for by meaningful work (R^2^ = .268). Next, a significant relationship between work engagement and job crafting was found (H2), *b* = .38, SE = .07, *p* < .001. Approximately 27% of the variance in job crafting was accounted for by work engagement (R^2^ = .273). Lastly, there was a significant relationship between job crafting and resilience (H3), *b* = .30, SE = .06, *p* < .001. Approximately 19% of the variance in resilience was accounted for by job crafting (R^2^ = .196). Thus, Hypothesis 1, 2 and 3 were confirmed by the data. In order to test whether work engagement and job crafting mediate the relationship between meaningful work and resilience (H4), a sequential mediation analysis was performed (Marco Hayes model 6; [66]). PROCESS uses a logistic regression-based path analytic framework for assessing the direct and indirect effects in conceptual mediation models [66]. First, the regression analysis of meaningful work on resilience, ignoring both mediators (work engagement and job crafting), was found to be significant (*b* = .20, SE = .70, *p* = .004). Second, the regression analysis of meaningful work on the first mediator work engagement was significant (*b* = .54, SE = .07, *p* < .001). Third, the regression analysis of work engagement on the second mediator job crafting was significant too (*b* = .38, SE = .07, *p* < .001). Fourth, the regression analysis of meaningful work on resilience, including both mediators (work engagement and job crafting), was found to be non-significant (*b* = .02, SE = .08, *p* = .745). Hence the results confirmed hypothesis 4. All in all, we concluded that work engagement and job crafting fully mediate the relationship between meaningful work and resilience (see Fig 2).

## Discussion

Over the past decade, research has constantly showed that teaching is an emotionally, physically, and intellectually challenging job [68–71]. As stated before, teachers’ resilience may be crucial to cope with the developments within Western society. Meaningful work may positively influence teachers’ resilience [7]. So far, relatively little was known about the processes through which meaningful work affects teacher’s resilience. The present study found support for a positive indirect relation between meaningful work and teacher’s resilience via teachers’ work engagement and subsequently their job crafting behavior.

### Theoretical contributions

The present study advances our understanding of the role that meaningful work play within schools and how meaningful work is linked to teachers’ resilience via work engagement and job crafting behavior. In this way, we advanced earlier research of Kim and colleagues [72] who focused primarily on the direct relationship between meaningful work and resilience. Our findings add to the literature on teachers’ resilience and are in line with research by Hansen [10] who stated that viewing ones job as meaningful can spark teachers resilience. In addition, the outcomes of this study are in line with the study by Van den Heuvel et al. [21], who suggested that employees’ understanding of how their work influences the outcomes of the organization can facilitate the development of positive attitudes (e.g. work engagement) and behaviors (e.g. job crafting). Hence, by showing the mediating mechanism of work engagement and job crafting, this study adds to the further understanding of the relationship between meaningful work and teachers’ resilience. As far as we know, this is the first study that revealed the positive impact of meaningful work on resilience via positive attitudes (e.g. work engagement) and behaviors (e.g. job crafting). Second, this study contributes to the literature on job crafting by shedding a light on the relationship between job crafting and teachers’ resilience. The study by Sammons et al., [4] showed that teachers working conditions may change in unpredictable ways, and depending on how they experience these changes, they will display resilience or be unable to cope with these changes. Our study showed that it may not only depend on how teachers’ experience change, but that their pro-active behavior via job crafting may lead to resilience. This is relevant because teachers’ resilience is needed to cope with challenges and changes they face in contemporary schools.

### Limitations and suggestions for future research

Although this study has it theoretical and practical contributions, several limitations of this study must be recognized. First, within the present study a cross-sectional dataset was used. Since the questionnaire was provided only at one point in time, it is not possible to make further predictions and to infer causality. Future research could examine if there are causal relationships between meaningful work, resilience, work engagement and job crafting, for example, by using longitudinal study designs. Second, the self-report nature of our data may potentially leads to self-report bias. By using this measurement method we cannot evade common method bias, possibly increasing the correlations among the study variables. Therefore, future research might explore additional, more objective ways to measure the variables used in this study [73], for example by using other ratings. Third, the study sample consisted of 174 mostly highly educated Dutch teachers. This may limit the generalizability of the outcomes of this study. With a small study sample it is difficult to generalize the results, therefore we recommend increasing the sample size in future studies. Future research may also try to replicate this study among teachers in different countries and cultures and among other occupational groups. Fourth, the sample of this study mainly included women. The study by Elizur [74] showed that women gave higher importance to meaningful work than men. Therefore, it is recommended that future research replicates this study among a study sample that contains an equal division of men and women. To conclude, in this study, the examined variables are measured from the perspective of the individual (e.g. personal context). Sammons et al., [4] established that resilience is a dynamic construct that is subjected to influences of environmental, work specific and personal context. Future research could consider the environmental and work specific context by for example, including the organizational climate in their studies.

### Practical implications

Based on the findings of this study, we offer several recommendations for school boards, principals and (HR)managers. Since teachers and schools have to cope with a continuously changing environment, it is important to recruit and retain teachers who experience their work to be meaningful. To do so, the experience of meaningful work may be the starting point for designing jobs and shaping HR policies and practices within schools. The cultivation of meaningful work is not only an important task for HR but also for management. School boards, principals and managers could deliberate influence how employees perceive their work. For example, senior management can play a crucial role in the cultivation of meaningful work within schools by clearly communication the goals, values, and contributions to society. In addition, they can show employees how the objectives of their work connect to their intrinsic values and beliefs. Initiating an ongoing dialogue about meaningful work may stimulate employees to continuously reflect upon their own work experience. Beside a more general approach,school boards, principals and managers can also influence teachers experience of meaningful work in a more direct way. To enhance teachers experience of meaningful work, managers should express appreciation for teachers contribution frequently, not merely during the annual performance cycle conversations. Further, managers should provide teachers with opportunities to craft their job. This present study showed that job crafting was positively related to teachers’ resilience. The study by Petrou et al., [52] suggested that even in the most stable work environments with detailed job descriptions and clear procedures, individuals can and will adjust the tasks they perform, and mobilize the resources they need to carry out their tasks successfully. Their results showed that employees might craft their jobs to create healthy and motivating working conditions. Through job crafting, employees may enhance their personal resources and their sustainable workability [75,76]. Thus, it is recommended that managers emphasize the importance of job crafting to the employees, as it may eventually affect their employability. Managers can do so by sharing examples of their own job crafting behavior with their employees and by sharing positive job crafting experiences of team members. In addition, organizations can also stimulate and enhance job crafting behavior among employees by offering job crafting interventions [51,76]. Lastly, school boards, principals and (HR)managers may also empower their teachers by offering training programs aimed at enhancing resilience. These type of training programs may support teachers to better cope with their changing work environment.

## Supporting information

S1 File Data meaningful work. (SAV)

## References

[1] Hargreaves A., & Fink D. (2006). Sustainable leadership. San Francisco, CA: Jossey-Bass.

[2] Dede C. (2010). Comparing frameworks for 21st century skills In: Bellanca J., & Brandt R.(Ed.), 21st century skills: Rethinking how students learn, 20, 51–76. Solution Tree Press.

[3] Voogt J., & Roblin N. P. (2012). A comparative analysis of international frameworks for 21st century competences: Implications for national curriculum policies. *Journal of curriculum studies*, 44(3), 299–321. 10.1080/00220272.2012

[4] Sammons P., Day C., Kington A., Gu Q., Stobart G., & Smees R. (2007). Exploring variations in teachers' work, lives and their effects on pupils: key findings and implications from a longitudinal mixed‐method study. *British educational research journal*, 33(5), 681–701. 10.1080/01411920701582264

[5] Hobfoll S. E., Johnson R. J., Ennis N., & Jackson A. P. (2003). Resource loss, resource gain, and emotional outcomes among inner city women. *Journal of personality and social psychology*, 84(3), 632 10.1037/0022-3514.84.3.632 12635922

[6] Windle G. (2011). What is resilience? A review and concept analysis. *Reviews in Clinical Gerontology*, 21(2), 152–169. 10.1017/S0959259810000420

[7] Fouché E., Rothmann S. S., & Van der Vyver C. (2017). Antecedents and outcomes of meaningful work among school teachers. *SA Journal of Industrial Psychology*, 43(1), 1–10. 10.4102/sajip.v43i0.1398

[8] Michaelson C., Pratt M. G., Grant A. M., & Dunn C. P. (2014). Meaningful work: Connecting business ethics and organization studies. *Journal of Business Ethics*,121(1), 77–90. 10.1007/s10551-013-1675-5

[9] Rosso B. D., Dekas K. H., & Wrzesniewski A. (2010). On the meaning of work: A theoretical integration and review. *Research in Organizational Behavior*, 30, 91–127. 10.1016/j.riob.2010.09.001

[10] Hansen D. T. (1995). The call to teach. New York: Teachers College Press.

[11] Littman-Ovadia H., & Steger M. F. (2010). Character strengths and well-being among volunteers and employees: Towards an integrative model. *The Journal of Positive Psychology*, 5, 419–430. 10.1080/17439760.2010.516765

[12] Steger M.F., Dik B.J., & Duffy R.D. (2012). Measuring meaningful work: The Work and Meaning Inventory (WAMI). *Journal of Career Assessment*, 20, 322–337. 10.1177/1069072711436160

[13] Steger M. F., Littman-Ovadia H., Miller M., Menger L., & Rothmann S. (2013). Engaging in work even when it is meaningless: Positive affective disposition and meaningful work interact in relation to work engagement. *Journal of Career Assessment*, 21(2), 348–361. 10.1177/1069072712471517

[14] Bakker A. B. (2011). An evidence-based model of work engagement. *Current Directions in Psychological Science*, 20(4), 265–269. 10.1177/09637214411414534

[15] Tims M., Bakker A. B., & Derks D. (2012). Development and validation of the job crafting scale. *Journal of vocational behavior*, 80(1), 173–186. 10.1016/j.jvb.2011.05.009

[16] Bakker A. B. (2010). Engagement and" job crafting": Engaged employees create their own great place to work In Albrecht S. L.(Ed.), New horizons in management. Handbook of employee engagement: Perspectives, issues, research and practice (pp. 229–244). Northampton, MA, US: Edward Elgar Publishing 10.4337/9781849806374.00027

[17] Tims M., Bakker A. B., & Derks D. (2013). The impact of job crafting on job demands, job resources, and well-being. *Journal of occupational health psychology*, 18(2), 230 10.1037/a0032141 23506549

[18] Berg J. M., Wrzesniewski A., & Dutton J. E. (2010). Perceiving and responding to challenges in job crafting at different ranks: When proactivity requires adaptivity. *Journal of Organizational Behavior*, 31(2–3), 158–186. 10.1002/job.645.

[19] Grant A. M., & Parker S. K. (2009). Redesigning work design theories: The rise of relational and proactive perspectives. Academy of Management Annals, 3, 317–375. 10.1080/19416520903047327

[20] Lu C. Q., Wang H. J., Lu J. J., Du D. Y., & Bakker A. B. (2014). Does work engagement increase person–job fit? The role of job crafting and job insecurity. *Journal of Vocational Behavior*, 84(2), 142–152. 10.1016/j.jvb.2013.12.004

[21] Van den Heuvel M., Demerouti E., Bakker A. B., & Schaufeli W. B. (2010). Personal resources and work engagement in the face of change. Contemporary occupational health psychology: Global perspectives on research and practice, 1, 124–150. 10.1002/9780470661550.ch7

[22] Berg J. M., Dutton J. E., & Wrzesniewski A. (2008). What is job crafting and why does it matter? Theory-to-practice briefing Ann Arbor, MI: School of Business, University of Michigan Ross Retrieved from http://positiveorgs.bus.umich.edu/wp-content/uploads/What-is-Job-Crafting-and-Why-Does-it-Matter1.pdf.

[23] Tims M., & Bakker A. B. (2010). Job crafting: Towards a new model of individual job redesign. *South African Journal of Industrial Psychology*, 36, 1–9. Retrieved from:http://www.scielo.org.za/scielo.php?script=sci_arttext&pid=S2071-07632010000200003&lng

[24] Wrzesniewski A., McCauley C., Rozin P., & Schwartz B. (1997). Jobs, careers, and callings: People’s relations to their work. *Journal of Research in Personality*, 31, 21–33. 10.1006/jrpe.1997.2162

[25] Meyers C. (2007). Industrial Psychology. New York: Garnsey Press.

[26] Frankl V. E. (1984). Man's search for meaning: An introduction to logo therapy. NewYork: Simon & Schuster.

[27] Cohen-Meitar R., Carmeli A., & Waldman D. A. (2009). Linking meaningfulness in the workplace to employee creativity: The intervening role of organizational Identification and positive psychological experiences. *Creativity Research Journal*, 21(4), 361–375. 10.1080/10400410902969910

[28] Sˇverko B., & Vizek-Vidovic´ V. (1995). Studies of the meaning of work: Approaches, models, and some of the findings In Super D. E. & Sˇ verko B. (Eds.), Life roles, values, and careers (pp. 3–21). San Francisco, CA: Jossey-Bass.

[29] Cascio W.F. (2003). Responsible restructuring: Seeing employees as assets, not costs. *Ivey Business Journal*, 68, 1–5.

[30] Martela F. and Pessi A.B. (2018, p.11). Significant Work Is About Self-Realization and Broader Purpose: Defining the Key Dimensions of Meaningful Work. *Front*. *Psychol*.9:363 10.3389/fpsyg.2018.00363 29632502 PMC5879150

[31] Anuradha M. V., Srinivas E. S., Singhal M., & Ramnarayan S. (2014). To work or not to work: Construction of meaning of work and making work choices. Vikalpa, 39(2), 7–20. 10.1177/0256090920140203

[32] Arnold K. A., Turner N., Barling J., Kelloway E. K., & McKee M. C. (2007). Transformational leadership and psychological well-being: The mediating role of meaningful work. Journal of Occupational Health Psychology, 12, 193–203. 10.1037/1076-8998.12.3.193 17638487

[33] Seibert S.E., Wang G. & Courtright S.H. (2011). Antecedents and consequences of psychological and team empowerment in organizations: a meta-analytic review. *Journal of Applied Psychology*, 96, 981–1003. 10.1037/a0022676 21443317

[34] Baumeister R. F., & Vohs K. D. (2002). The pursuit of meaningfulness in life In Snyder C. R. & Lopez S. J.(Eds.), *Handbook of positive psychology* (pp. 608–618). New York, NY, US: Oxford University Press.

[35] Neck C. P., & Milliman J. F. (1994). Thought self-leadership: Finding spiritual fulfilment in organizational life. *Journal of managerial psychology*, 9(6), 9–16. 10.1108/02683949410070151

[36] Pratt M. G., & Ashford B. E. (2003). Fostering meaningfulness in working and at work In Cameron K., Dutton J.E., & Quinn R.E.(Eds), *Positive organizational scholarship*:*Foundations of a new discipline* (pp. 308–327). San Fransisco: Berrett-Koehler.

[37] Holbeche L., & Springett N. (2004). In search of meaning at work Horsham, United Kingdom: Roffey Park Institute.

[38] May D. R., Gilson R. L., & Harter L. M. (2004). The psychological conditions of meaningfulness, safety and availability and the engagement of the human spirit at work. *Journal of Occupational and Organizational Psychology*, 77, 11–37. 10.1348/096317904322915892

[39] Milliman J., Czaplewski A. J., & Ferguson J. (2003). Workplace spirituality and employee Work attitudes: An exploratory empirical assessment. *Journal of Organizational Change Management*, 16(4), 426–447. 10.1108/09534810310484172

[40] Olivier A. L., & Rothmann S. (2007). Antecedents of work engagement in a multinational company. *SA Journal of Industrial Psychology*, 33(3), 49–56. 10.4102/sajip.v33i3.396

[41] Schaufeli W. B., Salanova M., González-Romá V., & Bakker A. B. (2002). The measurement of engagement and burnout: A two sample confirmatory factor analytic approach. Journal of Happiness studies, 3(1), 71–92. 10.1023/A:1015630930326

[42] Bakker A. B., Schaufeli W. B., Leiter M. P., & Taris T. W. (2008). Work engagement: An emerging concept in occupational health psychology. Work & Stre*ss*, 22(3), 187–200. 10.1080/02678370802393649 20103890

[43] Fairlie P. (2011). Meaningful work, employee engagement, and other key employee outcomes: Implications for human resource development. Advances in Developing Human Resources, 13(4):508–525. 10.1177/1523422311431679

[44] Nelson D. L., & Simmons B. L. (2003). Health psychology and work stress: A more positive approach In: Quick J.C. & Tetrick L.E. (Ed.), Handbook of occupational health psychology, (pp. 97–119). Washington, DC: American Psychological Association Retrieved from: http://psycnet.apa.org/record/2002-18427-000

[45] Van Wingerden J., & Van der Stoep J. (2017). The Role of Meaningful Work in Employees’ Work-Related and General Well-being. International Journal of Human Resource Studies, 7(4), 23–37. 10.5296/ijhrs.v7i4.11611

[46] Van Wingerden J., & Van der Stoep J. (2018). The motivational potential of meaningful work: Relationships with strengths use, work engagement, and performance. PloS one, 13(6), e0197599 10.1371/journal.pone.0197599 29897935 PMC5999113

[47] Schaufeli W. B., Taris T. W., Le Blanc P., Peeters M., Bakker A. B., & De Jonge J. (2001). Maakt arbeid gezond. Op zoek naar de bevlogen werknemer. De Psycholoog, 422–428. Retrieved from: https://www.wilmarschaufeli.nl/publications/Schaufeli/165n.pdf

[48] Schaufeli W. (2012). Work engagement: What do we know and where do we go?. *Romanian Journal of Applied Psychology*, 14(1), 3–10.

[49] Wrzesniewski A., & Dutton J. E. (2001). Crafting a job: Revisioning employees as active crafters of their work. *Academy of management review*, 26(2), 179–201. 10.5465/amr.2001.4378011

[50] Bakker A. B., & Demerouti E. (2014). Job demands–resources theory In: Chen P.Y. & Cooper C.L. (Ed.), Wellbeing: A complete reference guide, work and wellbeing (pp.1–28). New York: John Wiley & Sons 10.1002/9781118539415.wbwell019

[51] Van Wingerden J., Derks D., & Bakker A. B. (2017). The impact of personal resources and job crafting interventions on work engagement and performance. *Human Resource Management*, 56(1), 51–67. 10.1002/hrm.21758

[52] Petrou P., Demerouti E., Peeters M. C., Schaufeli W. B., & Hetland J. (2012). Crafting a job on a daily basis: Contextual correlates and the link to work engagement. *Journal of Organizational Behavior*, 33(8), 1120–1141. 10.1002/job.1783

[53] Hyvönen K., Feldt T., Salmela-Aro K., Kinnunen U., & Mäkikangas A. (2009). Young managers’ drive to thrive: A personal work goal approach to burnout and work engagement. *Journal of vocational Behavior*, 75(2), 183–196. 10.1016/j.jvb.2009.04.002

[54] Parker S. K., & Collins C. G. (2010). Taking stock: Integrating and differentiating multiple proactive behaviors. *Journal of Management*, 36(3), 633–662. 10.1177/0149206308321554

[55] Bindl U. K., & Parker S. K. (2010). 32 Feeling good and performing well? Psychological engagement and positive behaviors at work. Handbook of employee engagement: Perspectives, issues, research and practice, 385.

[56] Parker S. K., & Griffin M. A. (2011). Understanding active psychological states: Embedding engagement in a wider nomological net and closer attention to performance. *European Journal of Work and Organizational Psychology*, 20(1), 60–67. 10.1080/1359432X.2010.532869

[57] Luthans F. (2002). The need for and meaning of positive organizational behavior. *Journal of Organizational Behavior*, 23(6), 695–706. 10.1002/job.165

[58] Tugade M. M., & Fredrickson B. L. (2004). Resilient individuals use positive emotions to bounce back from negative emotional experiences. *Journal of personality and social psychology*, 86(2), 320 10.1037/0022-3514.86.2.320 14769087 PMC3132556

[59] Masten A.S. (2001). Ordinary magic: Resilience processes in development. *American Psychologist*, 56, 227–239. 10.1037//0003-066x.56.3.227 11315249

[60] Vogt K., Hakanen J. J., Brauchli R., Jenny G. J., & Bauer G. F. (2016). The consequences of job crafting: a three-wave study. *European Journal of Work and Organizational Psychology*, 25(3), 353–362. 10.1080/1359432X.2015.1072170

[61] Van Wingerden J., Bakker A. B., & Derks D. (2017). The longitudinal impact of a job crafting intervention. *European Journal of Work and Organizational Psychology*, 26, 107–119.

[62] Traag T. 2018 Leerkrachten basisonderwijs, Centraal Bureau voor Statistiek CBS, Den Haag, The Netherlands[Teachers within primary education, Central Bureau for Statistics.] Retrieved from: https://www.cbs.nl/nl-nl/achtergrond/2018/26/leerkrachten-in-het-basisonderwijs, july 16, 2019.

[63] Schaufeli W. B., Bakker A. B., & Salanova M. (2006). The measurement of work engagement with a short questionnaire: A cross-national study. *Educational and psychological measurement*, 66(4), 701–716. 10.1177/0013164405282471

[64] Luthans F., Avolio B. J., Avey J. B., & Norman S. M. (2007). Positive psychological capital: Measurement and relationship with performance and satisfaction. Personnel psychology, 60(3), 541–572. 10.1111/j.1744-6570.2007.00083.x

[65] Pallant J. (2013). SPSS survival manual: A step by step guide to data analysis using IBM SPSS (4th ed). Crows Nest, NSW: Allen & Unwin.

[66] Hayes A. F. (2017). Introduction to mediation, moderation, and conditional process analysis: A regression-based approach. Guilford Publications.

[67] Hayes A. F. (2012). PROCESS: A versatile computational tool for observed variable mediation, moderation, and conditional process modeling [White paper]. Retrieved from http://www.afhayes.com/public/process2012.pdf

[68] Biggs J. B. (2011). Teaching for quality learning at university: What the student does. McGraw-Hill Education (UK) SRHE and Open University Press.

[69] Danielson C. (2015). Talk about teaching!: Leading professional conversations. Corwin Press.

[70] Wassell B., & LaVan S. (2009). Tough transitions? Mediating beginning urban teachers’ practices through co teaching. *Cultural Studies of Science Education*, 4, 409–432. 10.1007/s11422-008-9151-8

[71] Zembylas M. (2010). Teachers’ emotional experiences of growing diversity and multiculturalism in schools and the prospects of an ethic of discomfort. *Teachers and Teaching*: *theory and practice*, 16(6), 703–716. 10.1080/13540602.2010.517687

[72] Kim M., Kim A. C. H., Newman J. I., Ferris G. R., & Perrewé P. L. (2018). The antecedents and consequences of positive organizational behavior: The role of psychological capital for promoting employee well-being in sport organizations. Sport Management Review, 22(1), 108–125. 10.1016/j.smr.2018.04.003

[73] Podsakoff P. M., MacKenzie S. B., Lee J., & Podsakoff N. P. (2013). Common method bias in behavioral research: A critical review of literature and recommended remedies. *Journal of Applied Psychology*, 88, 879–903. 10.1037/0021-9010.88.5.879 14516251

[74] Elizur D. (1994). Gender and work values: A comparative analysis. *The Journal of Social Psychology*, 134(2), 201–212. 10.1080/00224545.1994.9711383 8201817

[75] Van den Heuvel M., Demerouti E., & Peeters M. C. W. (2015). The job crafting intervention: Effects on job resources, self-efficacy, and affective well-being. *Journal of Occupational and Organizational Psychology*, 88, 511–532. 10.1111/joop.12128

[76] Van Wingerden J., Bakker A. B., & Derks D. (2017). Fostering employee well-being via a job crafting intervention. *Journal of Vocational Behavior*, 100, 164–174. 10.1016/j.jvb.2017.03.008

