# Correction: Meaningful work and resilience among teachers: The mediating role of work engagement and job crafting

**DOI:** 10.1371/journal.pone.0269347

**Published:** 2022-05-26

**Authors:** Sophie Groot Wassink, Jessica van Wingerden, Rob F. Poell

Sophie Groot Wassink should be included as first author in the author byline. Their affiliation is 1: the Department of Human Resource Studies, Tilburg University, Tilburg, The Netherlands. This author states they have no competing interests to declare.

The correct citation is: Groot Wassink S, Van Wingerden J, Poell RF (2019) Meaningful work and resilience among teachers: The mediating role of work engagement and job crafting. PLoS ONE 14(9): e0222518. https://doi.org/10.1371/journal.pone.0222518

The updated author contributions are as follows:

Conceptualisation: SGW, JvW

Data Curation: JvW

Formal analysis: JvW

Writing–original draft: SGW, JvW, RFP

Writing–review & Editing: JvW
